# Analysis of the Air Flow Generated by an Air-Assisted Sprayer Equipped with Two Axial Fans Using a 3D Sonic Anemometer

**DOI:** 10.3390/s120607598

**Published:** 2012-06-07

**Authors:** F. Javier García-Ramos, Mariano Vidal, Antonio Boné, Hugo Malón, Javier Aguirre

**Affiliations:** Superior Polytechnic School, University of Zaragoza, Huesca 22071, Spain; E-Mails: vidalcor@unizar.es (M.V.); anbone@unizar.es (A.B.); hml@unizar.es (H.M.); javier.aguirre@unizar.es (J.A.)

**Keywords:** sonic anemometer, 3D anemometer, sprayer, fan, air flow, air velocity

## Abstract

The flow of air generated by a new design of air assisted sprayer equipped with two axial fans of reversed rotation was analyzed. For this goal, a 3D sonic anemometer has been used (accuracy: 1.5%; measurement range: 0 to 45 m/s). The study was divided into a static test and a dynamic test. During the static test, the air velocity in the working vicinity of the sprayer was measured considering the following machine configurations: (1) one activated fan regulated at three air flows (machine working as a traditional sprayer); (2) two activated fans regulated at three air flows for each fan. In the static test 72 measurement points were considered. The location of the measurement points was as follow: left and right sides of the sprayer; three sections of measurement (A, B and C); three measurement distances from the shaft of the machine (1.5 m, 2.5 m and 3.5 m); and four measurement heights (1 m, 2 m, 3 m and 4 m). The static test results have shown significant differences in the module and the vertical angle of the air velocity vector in function of the regulations of the sprayer. In the dynamic test, the air velocity was measured at 2.5 m from the axis of the sprayer considering four measurement heights (1 m, 2 m, 3 m and 4 m). In this test, the sprayer regulations were: one or two activated fans; one air flow for each fan; forward speed of 2.8 km/h. The use of one fan (back) or two fans (back and front) produced significant differences on the duration of the presence of wind in the measurement point and on the direction of the air velocity vector. The module of the air velocity vector was not affected by the number of activated fans.

## 1. Introduction

Air assisted sprayers are the type of equipment most commonly used to apply pesticides in tree crops. A traditional air assisted sprayer is equipped with an axial ventilator placed in the back part of the tank. The air given off by the fan leads the droplets produced in the hydraulic nozzles up to the tree.

Air assisted sprayers must be regulated correctly in order to achieve a successful treatment. Four main factors affect the deposition efficiency: the nozzles type, the fluid pressure, the ground speed and the air volumetric flow rate. The combination of these parameters determines the applied volume rate which influences the quality of the treatment [1,2].

Both fluid pressure and nozzle type [3] determine the droplet size which affects spray movement to the target area and its deposition on the target [4,5]. In relation to the ground speed, deposition becomes more variable at higher speed and canopy locations farther from the sprayer discharge [6]. In general, low travel speed and high air output power improves air penetration [7].

Analyzing the effect of the air volumetric flow rate, large reductions in air volumetric flow rate can substantially reduce spray drift from axial fan sprayers without adversely affecting the variability of deposits on either leaf surface [8], though the relative mean amounts on the upper *versus* the lower leaf surface can change substantially. [9] concluded that the effect of air flow on spray penetration was significant beyond about 2 m depth in the tree. The provision for air flow adjustment on many commercial sprayers is generally inadequate to maintain an optimal spray distribution pattern across the broad range of orchard structures that exist on many commercial farms [8]. If the air velocities or volumes produced by the fans are low, the pesticides will reach the trees insufficiently. On the other hand, if these velocities or volumes are high, the pesticides are blown over and through the trees. Therefore to obtain an optimal coverage it is important to select an appropriate fan setting [10].

Currently, the technical manuals of the sprayers do not provide information on the characteristics of the air flow generated by the machine. In this sense, the air flow in the vicinity of the sprayer can be estimated using integrated computational fluid dynamics (CFD) [11]. However, experimental air velocity data are necessary to analyze the effect of the fan regulations on the treatment and to validate the CFD models.

The air flow generated by the sprayer fans can be characterized by using high precision anemometers such as sonic anemometers (2D and 3D) which are used to measure the velocity components at different heights, sections and distances from the machine [12,13]. The operational adjustment of the sprayers should be based on the knowledge of air discharge characteristics and fan type and thus on measurements of the generated air pattern [14]. A first step to analyze the air flow generated by a sprayer consists in analyzing the air flow patterns in a static way, with no motion of the sprayer and without considering crop interaction [15]. The static characterization of the air flow pattern is a useful tool to predict the sprayer performance in dynamic conditions, with the sprayer in motion at a selected forward speed. In this sense, several studies have shown the relation between static and dynamic measurements [14,15]. The static air flow pattern of the sprayers show a decrease in the air velocities values in comparison with dynamic tests [14], being more significant with increasing forward speeds of the sprayer. The combination of the air output flow and the forward travel speed, expressed as the available flow per meter in travel direction, is one of the sprayer's operating properties. For hedgerow plantings (about 2 m tall), 2.8 m^3^/m would be sufficient to penetrate the trees at forward speeds of up to 11 km/h.

The air flow pattern depends on the number and disposition of the sprayer fans. In recent years, some manufacturers have designed sprayers equipped with several fans [11,16]: two fans placed in parallel behind the tank, two fans placed in line behind the tank, a fan placed in front of the tank and one behind, two rear fans at different heights, and so on. Thus, the interaction of the air flows generated by the fans improves the penetration of the chemical product in the crop. In [16] the authors carried out field tests with a sprayer equipped with two reversed rotation axial fans, one placed in the back part of the tank and the other one placed in the front part. Results showed a better product penetration in the crop compared to traditional sprayers. Progress is needed in characterizing the air flow generated by these new machine designs to rigorously determine the advantages obtained by these sprayers in the application of the plant protection products.

## 2. Experimental Section

The objective of this research has been to characterize the air flow generated by an air-assisted sprayer equipped with two reversed rotation axial fans by measuring the air velocity with a 3D sonic anemometer. A second goal has been the comparative analysis of the air flow generated by using two fans (front and back) *versus* a single fan (back). Most of the measurements have been taken in a static way to characterize the sprayer regardless of the working conditions in field.

### 2.1. Air Volumetric Flow Rate of the Sprayer Fans

**Figure 1.**
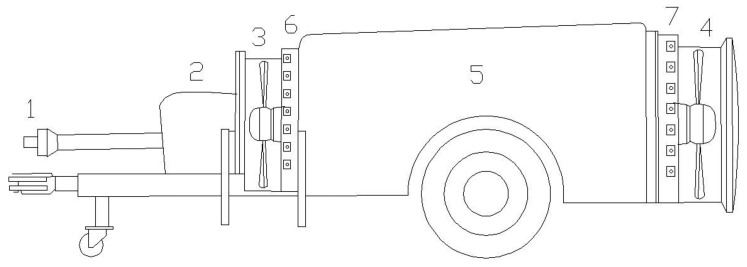
Air assisted sprayer equipped with two reversed rotation axial fans. 1 PTO; 2 Pump; 3 Front fan; 4 Back fan; 5 Tank; 6 Front nozzles; 7 Back nozzles.

The sprayer analyzed was developed by Gar Melet S.L (Huesca, Spain). The sprayer (Figure 1) is equipped with two reversed rotation axial fans [16], one placed in the back part of the tank and the other one placed in the front part. Both fans turn in opposite senses to guarantee a uniform distribution of the chemical product to be applied. The diameter of the front fan was 800 mm and that of the back fan 830 mm.

**Figure 2.**
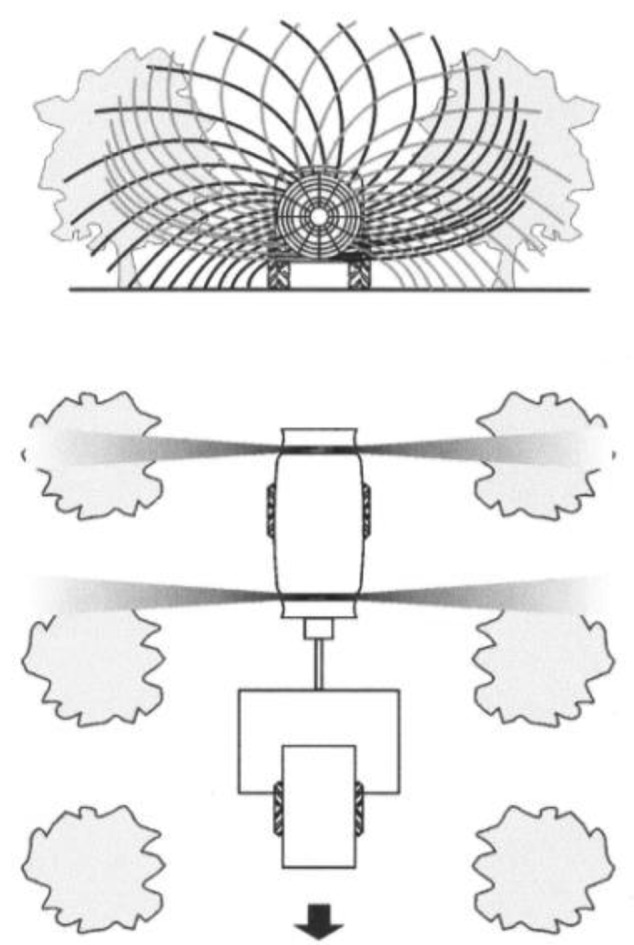
Air flow produced by the front and back fans.

The fan placed in the front part of the tank takes the air ahead and directs it in a radial form towards the trees, whereas the back fan takes the air behind. As it is shown in Figure 2, the rotation sense of both fans is opposite. The objective of this design is to achieve a similar air flow on the left and right sides of the treated rows.

Back and front fans can be regulated, in a range from 1 to 5, to supply different air flows by turning the fan blades. In this sense, the air flows were measured according to [17] considering three positions of the fan blades (1.5; 3; 5). The air flow corresponding to the back fan was measured at the fan's inlet, using a TESTO 0635 1041 hot wire anemometer (accuracy 0.03 m/s; range 0–20 m/s). The air flow rate of the front fan was measured at the outlet of the fan because of the presence of the power take off (PTO) of the tractor. With this aim, a Prandtl tube was used. Measurements were carried out with the PTO working at 540 rpm.

### 2.2. Static Characterization of the Air Flow Generated by the Sprayer

**Figure 3.**
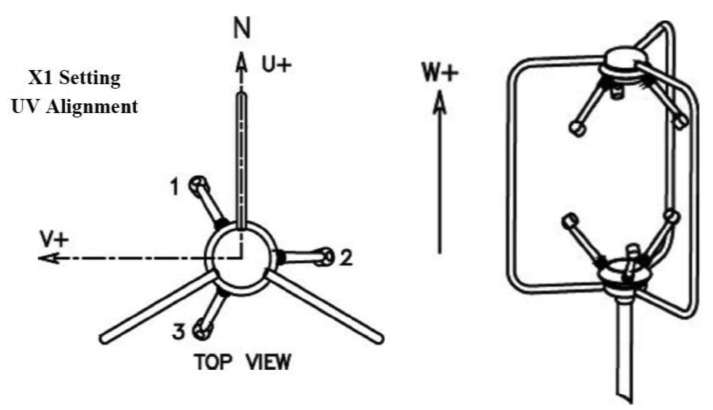
Configuration of the 3D sonic anemometer axes.

The velocity of the air generated by the sprayer was measured in absence of wind using a WindMaster (Gill Instruments) 3D sonic anemometer (Figure 3).

The sonic anemometer accuracy was of 1.5% (for win speed up to range maximum) with an air velocity range of 0 to 45 m/s and a resolution of 0.01 m/s. Data of air velocity were recorded in Cartesian coordinates (U, V, W) according to the axes of Figure 3, at a frequency of 1 Hz, using the Daqlink v1.2.2.1 software (Fourier Systems Ltd.).

**Figure 4.**
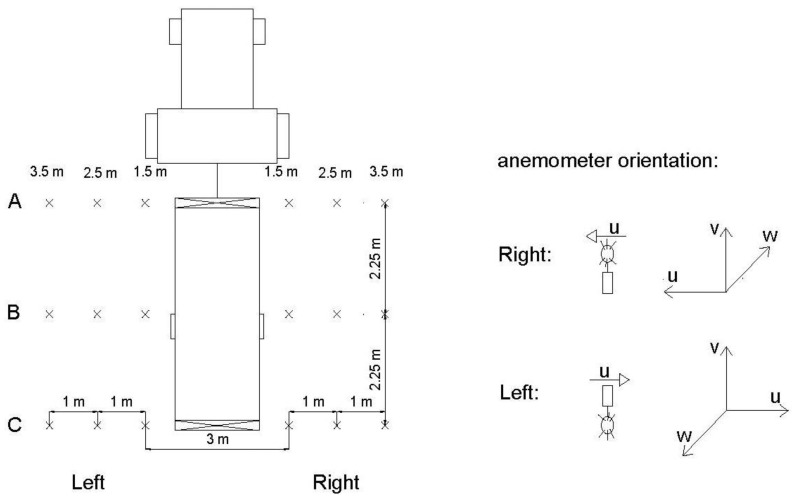
Outline of the measuring points of the static analysis.

Measurements were carried out with the sprayer working in a static way, establishing three regulations of the fans (1.5, 3 and 4.5) and consequently several air volumetric flow rates. The air velocity was measured, based on previous research [14], in three sections of the sprayer: A, B and C (Figure 4). For each section, measurements were made on both sides of the machine at 1.5, 2.5 and 3.5 m from the centre of the sprayer for several heights: 1, 2, 3 and 4 m.

The sprayer was regulated to analyze six working conditions linked to six different air flows with the goal of analyzing the effect of using an only fan (back fan; traditional sprayers) in comparison with the use of two fans (front and back; new design of sprayer):

- Front fan in position 1.5 and back fan in position 1.5.
- Front fan in position 3 and back fan in position 3.
- Front fan in position 4.5 and back fan in position 4.5.
- Back fan in position 1.5.
- Back fan in position 3.
- Back fan in position 4.5.

For each measuring point and sprayer setting, data were recorded during 60 s. The anemometer orientation for the measurements is shown in Figure 4.

### 2.3. Dynamic Measurements of the Air Flow Generated by the Sprayer

**Figure 5.**
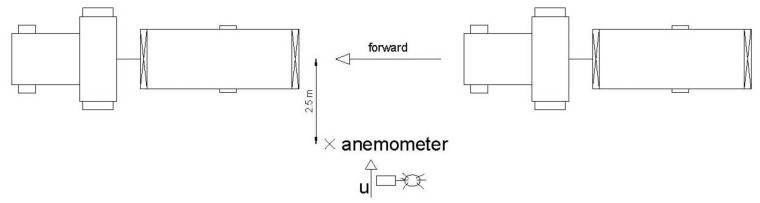
Outline of the measurement point of the dynamic analysis.

A dynamic test was performed to analyze the air flow generated by the sprayer in field working conditions (Figure 5). The air velocity was measured with the Windmaster sonic anemometer at 2.5 m from the centre of the sprayer considering several heights: 1, 2, 3 and 4 m.

The sprayer was regulated to analyze two working conditions with the goal of comparing the air velocity data with those of the static test:

- Front fan in position 3 and back fan in position 3.
- Back fan in position 3.

The test was performed in absence of wind at a forward speed of 2.8 km/h with the tractor PTO at 540 rpm. For each measuring configuration three replicates were performed.

## 3. Results and Discussion

### 3.1. Air Volumetric Flow Rate of the Sprayer Fans

The air flow provided by front and back fans was quantified from the inlet air velocity (rear fan) and outlet air velocity (front fan). Table 1 shows the air flow data.

### 3.2. Static Characterization of the Air Flow Generated by the Sprayer

In a first step, an univariate test of significance (Statistica 7, Statsoft Inc., Tulsa, OK, USA) was developed to evaluate the effect of the independent variables on the air velocity (considered as the dependent variable). In this sense, the independent variables were: activated fan (back, front); air flow (fan blades position 1.5, 3, 4.5); measuring section (A, B, C); sprayer side (left, right); distance of the measuring point (1.5 m, 2.5 m, 3.5 m); height of the measuring point (1 m, 2 m, 3 m, 4 m). The air velocity values corresponded to the resulting absolute velocity of the three Cartesian coordinates (module of the velocity vector).

Table 2 shows the results of the test where the variables that significantly affected the value of air velocity are highlighted in red. Section, height and distance were the most influential variables, which is logical since these variables refer to the position of the measuring point. However the sprayer side did not affect the air velocity significantly, which shows a symmetrical distribution of the air at both sides of the machine.

With reference to the two variables related to the sprayer regulation (activated fan and fan blades position), the number of activated fans had a bigger influence on the air velocity values than the air flow. In a first analysis, this fact reflects that the new design of sprayer (back and front fans) produces bigger differences in the air flow pattern in comparison with traditional designs (back fan).

**Figure 6.**
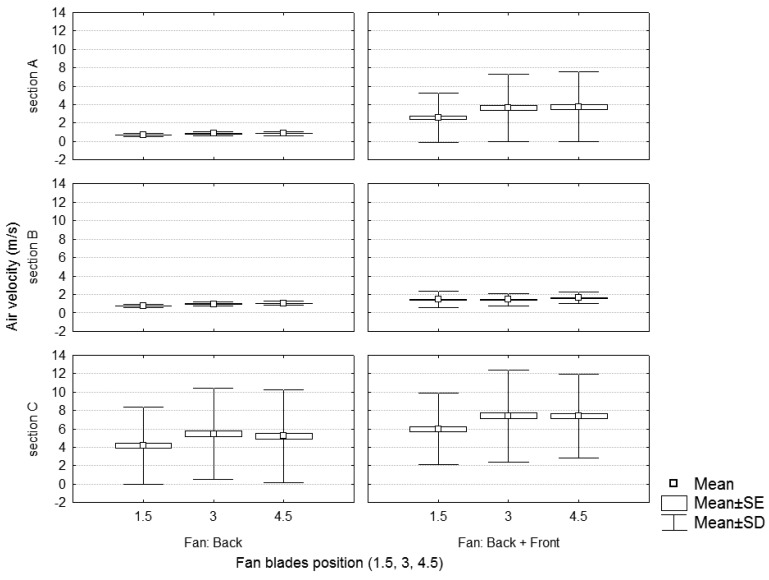
Static test. Air velocity values in the sample sections depending on the number of active fans and the air flow generated.

**Figure 7.**
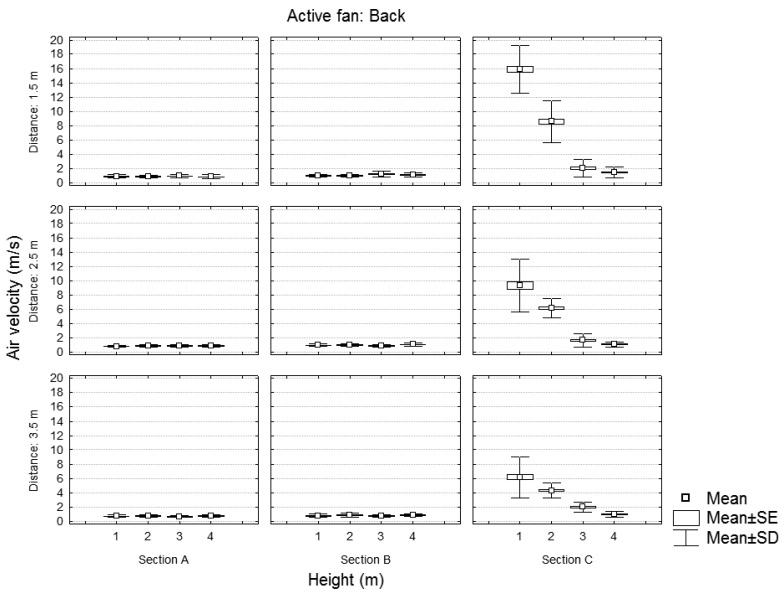
Static test of the sprayer with the back fan powered. Air velocity values for each section in function of the height and distance of the measurement point.

Figures 6 and 7 show the air velocity values according to the position (distance and height) of the measurement point for the case of one or two activated fans, respectively. Data are grouped by different fan blade positions. Results of Figure 6 (one fan) are concordant with those obtained by [15] who showed, for a static test of a traditional sprayer, a decrease in the air velocities with the distance and the height, and an increase with the air flow.

A Tukey test was developed to analyze the effect of the sprayer regulation on the air velocity. The independent variables considered were the activated fan (back, front) and the fan blades position (1.5, 3, 4.5). Results are shown in Table 3. Comparing the use of one fan (back) or two fans (back and front), there were significant differences in the air velocity, mainly due to the presence of wind in section A when both fans were used. Considering the sprayer working with the back fan, in a similar way to a traditional sprayer, there were no significant differences in the air velocity due to the fan settings (fan blades' position 1.5, 3 and 4.5). However, considering the sprayer working with the two fans (back and front) there were significant differences between the fan blades' position 1.5 and the fan blades' positions 3 and 4.5.

Figure 6 shows the air velocity values in the working sections (A, B and C) in function of the fan blades' position and the number of activated fans. It can be concluded that using two fans (front and back) at the lowest air flow (fan blades in position 1.5) produces higher air velocities, at any section, than using a single fan (back fan) at maximum air flow rate (fan blades in position 4.5). This fact is logically predictable in section A, considering that the machine, in the case of working with both fans, produces wind in the area nearest to the front fan. However the air velocity is also kept higher for sections B and C, showing that the new sprayer design allows for higher air velocities in the spray area in comparison with traditional sprayers.

**Figure 8.**
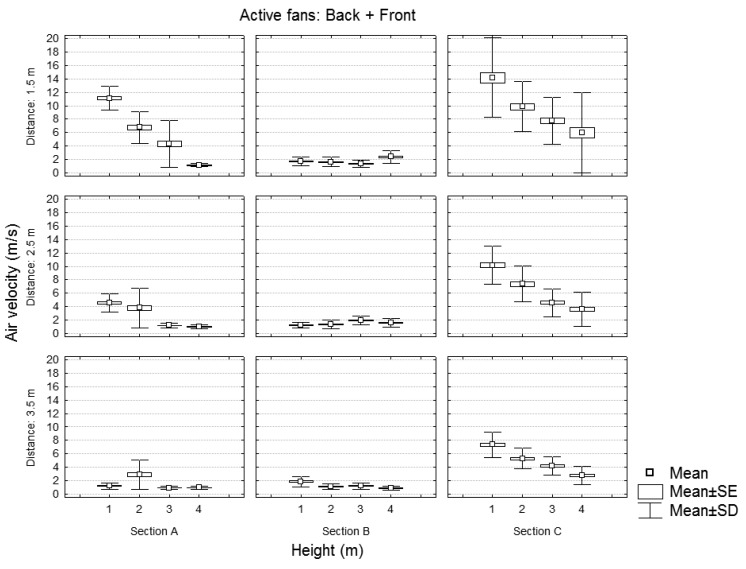
Static test of the sprayer with the back and front fans powered. Air velocity values for each section in function of the height and distance of the measurement point.

Figures 7 and 8 show, for any distance and height of the measuring point, the air velocity values recorded in the three sections (A, B, and C) for the case of one or two activated fans. As expected, the air velocity values decreased with height and distance. The variation in air velocity values was similar to that obtained by [14], who concluded that, considering a static test with a traditional sprayer, air velocities at a distance of 3.5 m were half as high as at 1.75 m.

Considering the sprayer working with the two fans activated, the air velocities registered in section C, corresponding to the front fan, explain the difference in the global air flow generated by the sprayer. In this sense, when the machine is working with two activated fans (front and back), there would be predictably an increase in the crop area affected by the wind generated by the sprayer per unit of time because more sections would receive air flow, and, besides, the air velocity values would be higher. The consequence would be an increase in the vegetation movement and a better penetration and deposition of the sprayed product. This would explain the results obtained by [16] who demonstrated that, with this new spray design and considering similar doses of applied product (l/ha), significant increases were obtained in the deposition of chemical product on the crop when two fans were used.

**Figure 9.**
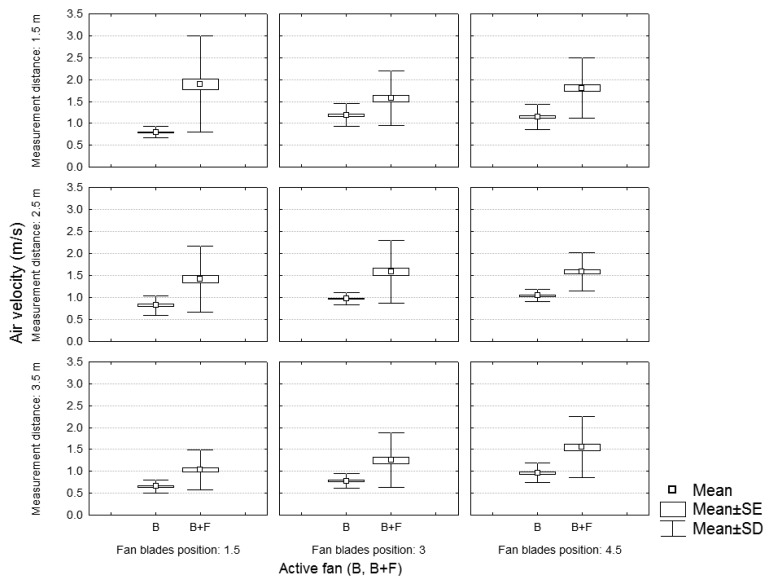
Static test. Air velocity values in the section B in function of the number of active fans and the distance of measurement, considering different fan blades positions.

The increase in the air velocity in section B reflects the interaction between the air flows generated by both fans more clearly. Figure 9 shows, for any distance, the air velocities recorded in section B were higher in the case of two activated fans.

The symmetry of the air flow generated by the sprayer was analyzed by developing a Tukey test for the variable air velocity, considering as independent variables the active fans (back, front), the measuring distance (1.5, 3, 4.5) and the machine side (left, right). According to Table 4, there were not significant differences in the air velocities registered at both sides of the sprayer.

This fact has been analyzed by other researchers [10], who concluded that, for most sprayers, at least a small difference in the air field between the left and right side can be observed. In this sense, the design of the sprayer with the fans turning in opposite directions produced a symmetrical distribution of the air flow.

**Figure 10.**
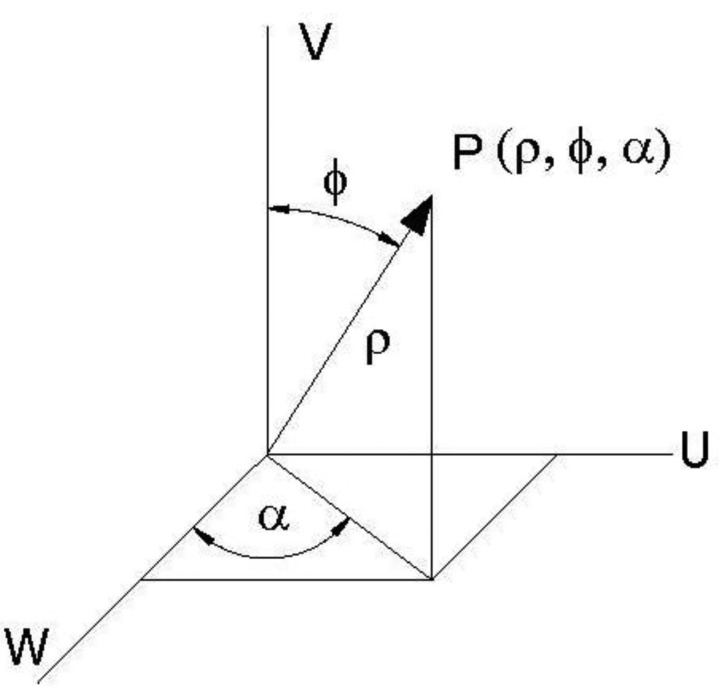
Polar coordinates of the velocity vector measured by the 3D sonic anemometer.

**Figure 11.**
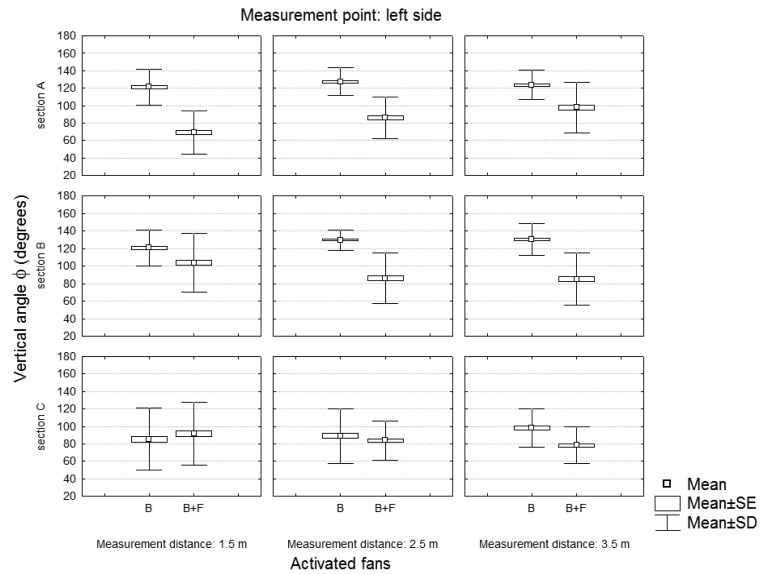
Vertical angle of the velocity vector according to the number of active fans and the measurement section. Values of the left working side.

**Figure 12.**
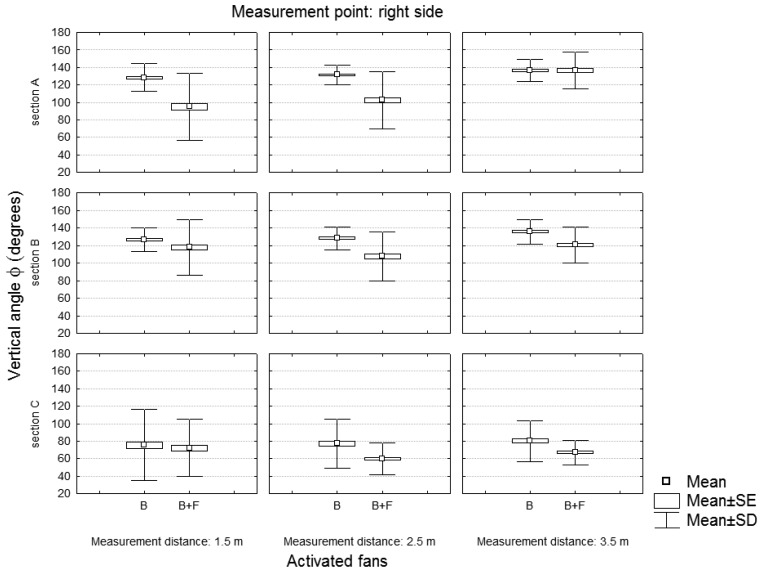
Vertical angle of the velocity vector according to the number of active fans and the measurement section. Values of the right working side.

The air flow characteristics were also studied by analyzing the vertical angle φ of the velocity vector expressed in polar coordinates (Figure 10). Figures 11 and 12 show the variation of the vertical angle values according to the working side analyzed (left or right), the number of active fans and the location of the measuring point.

Table 5 shows the results of a univariate test of significance developed to analyze the first-order effects of the independent variables on the dependent variable (vertical angle of the velocity vector). Variables that significantly affected the value of the vertical angle of the air velocity vector are highlighted in red. Activated fans and section were the most influential variables, highlighting the influence of the use of two fans in the variation of the direction of the air velocity vector.

### 3.3. Dynamic Measurements of the Air Flow Generated by the Sprayer

**Figure 13.**
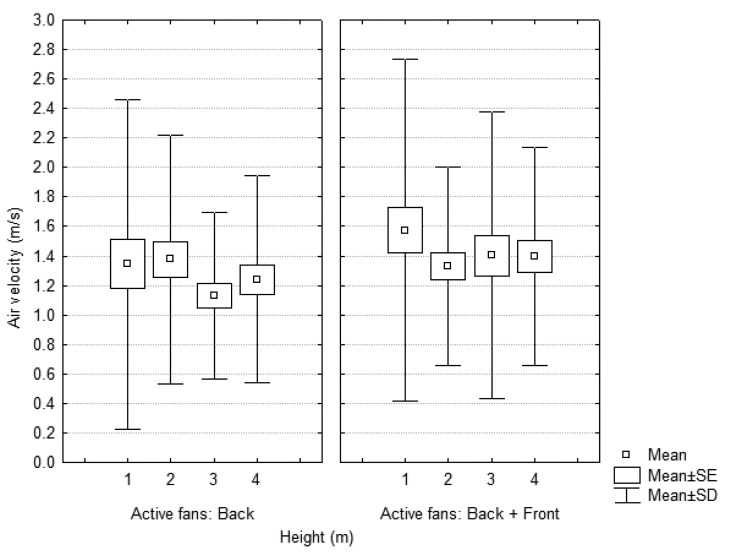
Dynamic test. Air velocity values in function of the number of active fans and the height of measurement. Measurement conditions: distance = 2.5 m; fan blades position = 3.

A dynamic test was developed to analyze the air flow pattern under field conditions. The measurement point was set at a distance of 2.5 m and the fan blades of the sprayer were set in position 3 with the goal of validating the static test results. The effect of the number of active fans (back, back+front) on the air velocity was analyzed by developing a univariate test of significance (Figure 13). In this sense, the number of active fans did not affect the air velocity significantly. This fact was predictable because the range of air velocities obtained with a single fan (back) was similar to that obtained with two (back+front) considering that the measuring point was located in a unique section where the total range of wind values were registered (in comparison with the static test where the differences between air values in the measurement sections varied according to the number of activated fans). Besides, the air velocity decreased significantly with the height of the measurement point.

**Figure 14.**
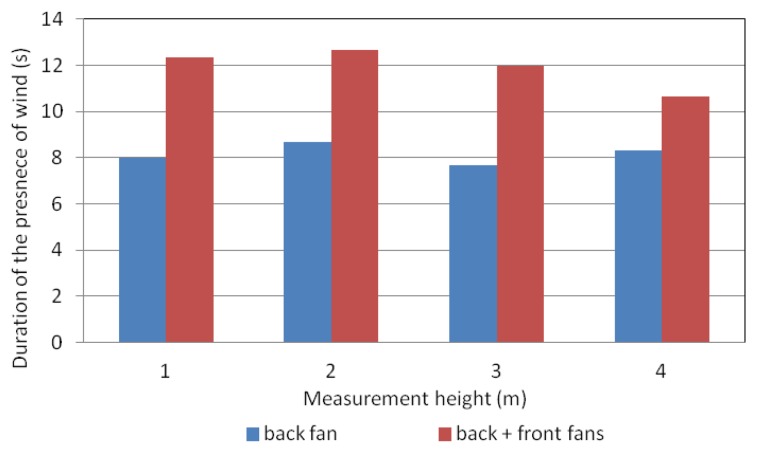
Dynamic test. Duration of the presence of wind in the machine vicinity. Measurement conditions: distance = 2.5 m; fan blades position = 3; heights = 1, 2, 3 and 4 m.

Although the air velocity values were similar considering one or two fans, the use of two fans implied a larger time of presence of wind in the sprayer vicinity. Figure 14 shows the duration of the period of time in which the anemometer registered wind values. As a mean value, the use of two fans produced a duration of the period of wind 45% higher than the use of a single fan. This fact would validate the data obtained in the static test in the sense that the use of two fans would produce an increase in the vegetation movement and a better penetration and deposition of the sprayed product.

**Figure 15.**
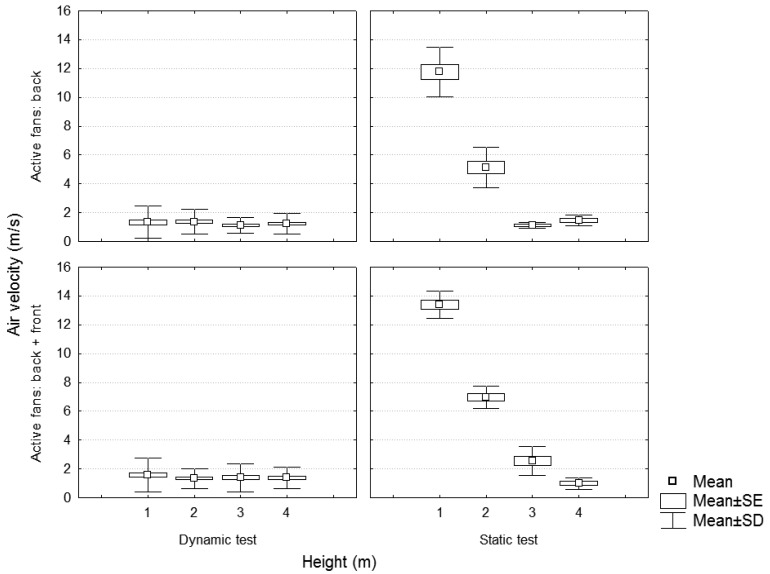
Dynamic *versus* static test. Fan blades position = 3. Air velocity values in function of the measurement height and the number of active fans. Static values measured in section C.

Considering static and dynamic tests (Figure 15), the results showed a decrease in the air velocity values of the dynamic test, giving similar values for measurement heights over 3 m. These results are concordant with those of [15], who concluded that considering a traditional sprayer and a specific distance of measurement, the air velocities values at different heights were higher for the static test, and that these differences were less relevant for values measured at heights over 3 m. These results are also concordant with those of [14], who described a decrease in the air velocities values of the static tests in comparison with dynamic tests, being more significant with increasing forward speeds.

**Figure 16.**
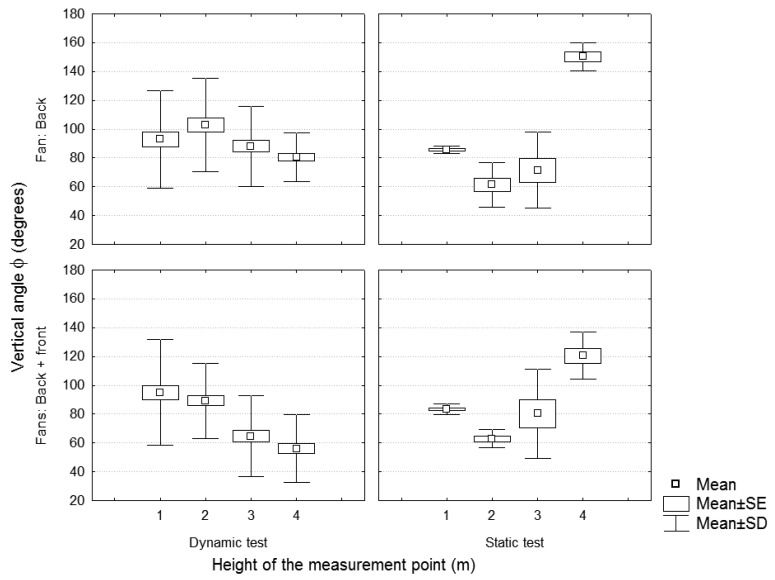
Dynamic *versus* static test. Fan blades' position = 3. Vertical angle of the velocity vector in function of the measuring height and the number of activated fans. Static values measured in section C.

The vertical angles φ of the velocity vector (Figure 16), in the same way as in the static test, were lower when two fans were used.

The variations in the vertical angle values were lower considering the dynamic test. However no significant differences were found between the vertical angles comparing the dynamic and the static tests.

## 4. Conclusions

The use of 3D anemometers to characterize the air velocities generated by a sprayer equipped with two fans has allowed the results obtained to explain the differences found in the spray deposition in field conditions according to the number of activated fans and the air flow rate of each fan.

When the machine is working with two activated fans (front and back), there would be predictably an increase in the crop area affected by the wind generated by the sprayer per unit of time because more sections would receive air flow, and besides, the air velocity values would be higher.

Considering the static test, the use of one fan (back) or two fans (back and front) produces significant differences on the module and direction of the air velocity vector.

Considering the dynamic test, the use of one fan (back) or two fans (back and front) produces significant differences in the duration of the presence of wind at the measurement point and on the direction of the air velocity vector. The module of the air velocity vector is not affected by the number of activated fans.

## Figures and Tables

**Table 1.** Air flows of the back and front fans of the sprayer.

**Fan**	**Blade setting**	**Air flow (m^3^/h)**

Back	1.5	27,373
	3	38,654
	4.5	42,831

Front	1.5	21,649
	3	32,194
	4.5	35,375

**Table 2.** Univariate test of significance for the velocity air in the static test.

**Variable**	**SS**	**Degr. F**	**MS**	**F**	***p***

Intercept	40,790.51	1	40,790.51	95,289.07	0.000
{1}Fan (back, front)	3,118.50	1	3,118.50	7,285.01	0.000
{2}Fan blades position (1.5, 3, 4.5)	472.89	2	236.44	552.35	0.000
{3}Section (A, B, C)	18,269.40	2	9,134.70	21,339.21	0.000
{4}Distance (1.5 m, 2.5 m, 3.5 m)	3,692.68	2	1,846.34	4,313.16	0.000
{5}Height (1 m, 2 m, 3 m, 4 m)	7,488.80	3	2,496.27	5,831.42	0.000
{6}Side (left, right)	0.00	1	0.00	0.00	1.000

**Table 3.** Tukey HSD test; dependent variable: air velocity (m/s). Independent variables: fan (back B; front F); fan blades position (1.5, 3, 4.5).

**Probabilities for *Post Hoc* Tests Error: Between MS = 13.662, df = 4,314.0**								

	**Fan**	**Fan blades position**	**{1}**	**{2}**	**{3}**	**{4}**	**{5}**	**{6}**

{1}	B	1.5		0.062871	0.123359	0.000020	0.000020	0.000020
{2}	B	3	0.062871		0.999825	0.000063	0.000020	0.000020
{3}	B	4.5	0.123359	0.999825		0.000031	0.000020	0.000020
{4}	B+F	1.5	0.000020	0.000063	0.000031		0.000189	0.000046
{5}	B+F	3	0.000020	0.000020	0.000020	0.000189		0.998916
{6}	B+F	4.5	0.000020	0.000020	0.000020	0.000046	0.998916	

**Table 4.** Tukey HSD test; dependent variable: air velocity (m/s). Independent variables: fan (back B; front F); distance (1.5 m, 2.5 m, 3.5 m); sprayer side (left L, right R).

**Probabilities for Post Hoc Tests Error: Between MS = 12.788, df = 4308.0**														

	**Fan**	**Distance (m)**	**Side**	**{1}**	**{2}**	**{3}**	**{4}**	**{5}**	**{6}**	**{7}**	**{8}**	**{9}**	**{10}**	**{11}**

**1**	B	1.5	L		1.0000	0.1261	0.0099	0.0000	0.0000	0.0000	0.0000	0.7535	0.6058	0.9873
**2**	B	1.5	R	1.0000		0.2712	0.0313	0.0001	0.0000	0.0000	0.0000	0.5220	0.3712	0.9991
**3**	B	2.5	L	0.1261	0.2712		0.9997	0.5426	0.5221	0.0000	0.0000	0.0000	0.0000	0.8590
**4**	B	2.5	R	0.0099	0.0313	0.9997		0.9621	0.9563	0.0000	0.0000	0.0000	0.0000	0.3263
**5**	B	3.5	L	0.0000	0.0001	0.5426	0.9621		1.0000	0.0000	0.0000	0.0000	0.0000	0.0048
**6**	B	3.5	R	0.0000	0.0000	0.5221	0.9563	1.0000		0.0000	0.0000	0.0000	0.0000	0.0043
**7**	B+F	1.5	L	0.0000	0.0000	0.0000	0.0000	0.0000	0.0000		0.8453	0.0000	0.0000	0.0000
**8**	B+F	1.5	R	0.0000	0.0000	0.0000	0.0000	0.0000	0.0000	0.8453		0.0000	0.0000	0.0000
**9**	B+F	2.5	L	0.7535	0.5220	0.0000	0.0000	0.0000	0.0000	0.0000	0.0000		1.0000	0.0752
**10**	B+F	2.5	R	0.6058	0.3712	0.0000	0.0000	0.0000	0.0000	0.0000	0.0000	1.0000		0.0392
**11**	B+F	3.5	L	0.9873	0.9991	0.8590	0.3263	0.0048	0.0043	0.0000	0.0000	0.0752	0.0392	
**12**	B+F	3.5	R	0.6907	0.8792	0.9987	0.8504	0.0717	0.0661	0.0000	0.0000	0.0051	0.0021	0.9997

**Table 5.** Univariate test of significance for the vertical angle of the air velocity vector in the static test.

**Variable**	**SS**	**Degr. F**	**MS**	**F**	***p***

Intercept	45,817,737	1	45,817,737	63,387.53	0.000000
{1}Fan (back, front)	490,843	1	490,843	679.07	0.000000
{2}Fan blades position (1.5, 3, 4.5)	20,108	2	10,054	13.91	0.000001
{3}Section (A, B, C)	1,160,429	2	580,214	802.71	0.000000
{4}Distance (1.5 m, 2.5 m, 3.5 m)	44,273	2	22,137	30.63	0.000000
{5}Height (1 m, 2 m, 3 m, 4 m)	152,066	3	50,689	70.13	0.000000
{6}Side (left, right)	27,813	1	27,813	38.48	0.000000
Error	3,113,906	4308	723		
